# Beyond the interferon score: neurofilament light chain and glial fibrillary acidic protein capture immune-mediated neuroinjury and response to JAK inhibition in Aicardi–Goutières syndrome

**DOI:** 10.3389/fimmu.2026.1782352

**Published:** 2026-04-15

**Authors:** Lisa Wege, Christian Klemann, Sandy Siegert, Annette Bley, Sarah Koss, Anne Koy, Thorsten Langer, Franziska Dunst, Barbara Plecko, Martin Fleger, Fabian Speth, Tim Niehues, Peter Kaufmann, Jurek Schultz, Mohammed Attia, Johannes Stoffels, Beata Wolska-Kuśnierz, Roman Rolke, Gabriele Reichelt, Friederike Blankenburg, Jürgen Brunner, Katja Akgün, Christine Wolf, Min Ae Lee-Kirsch

**Affiliations:** 1 Department of Pediatrics, Medizinische Fakultät Carl Gustav Carus, Technische Universität Dresden, Dresden, Germany; 2 Department of Pediatric Immunology, Rheumatology and Infectiology, Hospital for Childrens and Adolescents, University of Leipzig, Leipzig, Germany; 3 Department of Pediatrics and Adolescent Medicine, Division of Pediatric Pulmonology, Allergology and Endocrinology, Medical University of Vienna, Vienna, Austria; 4 University Children’s Hospital, University Medical Center Hamburg-Eppendorf, Hamburg, Germany; 5 Department of Pediatrics and Centre for Rare Diseases, Faculty of Medicine and University Hospital of Cologne, University of Cologne, Cologne, Germany; 6 Department of Neuropediatrics and Muscle Disorders, Children’s Hospital, Faculty of Medicine, University of Freiburg, Freiburg, Germany; 7 Department of Pediatrics, Division of General Pediatrics, Medical University of Graz, Graz, Austria; 8 Department of Pediatrics, State Hospital of Bregenz, Bregenz, Austria; 9 Department of Pediatric Rheumatology, Pediatric Bone Marrow Transplantation and Immunology Unit, Center for Obstetrics and Pediatrics, University Medical Center Hamburg-Eppendorf, Hamburg, Germany; 10 Department of Pediatrics, Helios Klinik Krefeld, Krefeld, Germany; 11 Department of Pediatrics, Gesundheitsbezirk Bozen, Südtiroler Sanitätsbetrieb, Bozen, Italy; 12 Department of Pediatric Surgery, Medizinische Fakultät Carl Gustav Carus, Technische Universität Dresden, Dresden, Germany; 13 Vinzentius Krankenhaus Landau, Landau, Germany; 14 Division of Neuropediatrics and Integrated Health Care, Department of Pediatrics and Adolescent Medicine, KJF Klinikum Josefinum, Augsburg, Germany; 15 Department of Immunology, Children’s Memorial Health Institute, Warsaw, Poland; 16 Department of Palliative Medicine, Medical Faculty Rheinisch-Westfälische Technische Hochschule (RWTH) Aachen University, Aachen, Germany; 17 Department of Pediatrics, University Medical Center Mainz, Mainz, Germany; 18 Pediatric Rheumatology, Olgahospital, Stuttgart, Germany; 19 Pediatric Rheumatology, Department of Pediatrics, Medizinische Universität Innsbruck, Innsbruck, Austria; 20 Department of Medicine, Faculty of Medicine and Dentistry, Danube Private University, Krems, Austria; 21 Multiple Sklerose Zentrum Dresden, Medizinische Fakultät Carl Gustav Carus, Technische Universität Dresden, Dresden, Germany; 22 German Center for Child and Adolescent Health (DZKJ), partner site Leipzig/Dresden, Dresden, Germany; 23 University Center for Rare Diseases, Medizinische Fakultät Carl Gustav Carus, Technische Universität Dresden;, Dresden, Germany

**Keywords:** Aicardi-Goutières syndrome, biomarker, immune-mediated neurodegeneration, inflammation, interferon, JAK inhibition, neurofilament light chain, glial fibrillary acidic protein

## Abstract

**Background and objective:**

Aicardi–Goutières syndrome (AGS) is a prototypical type I interferon–driven neuroimmunological disorder in which immune-mediated inflammation causes progressive brain injury. Targeted immunosuppression with Janus kinase (JAK) inhibitors has shown clinical benefit, yet neurological outcomes remain difficult to quantify, and the interferon (IFN) score poorly reflects neuroaxonal damage. We investigated whether plasma neurofilament light chain (pNfL) and glial fibrillary acidic protein (pGFAP) serve as sensitive biomarkers of neurological involvement and response to immunosuppressive therapy.

**Methods:**

Plasma samples from 55 patients with genetically confirmed AGS and 55 age- and sex-matched healthy controls were analyzed using single molecule array assays. pNfL and pGFAP levels were assessed in relation to age, clinical disease severity, IFN score, and treatment with JAK inhibitors. Longitudinal samples before and during therapy were available from 11 patients.

**Results:**

Treatment-naïve AGS patients exhibited significantly elevated pNfL and pGFAP levels compared with controls, and biomarker concentrations correlated with clinical disease severity. pNfL and pGFAP were strongly correlated with each other but showed only weak to moderate associations with the IFN score. Longitudinal within-patient analyses demonstrated significant declines in pNfL and pGFAP following initiation of JAK inhibitor therapy, whereas the IFN score remained unchanged.

**Discussion:**

pNfL and pGFAP are sensitive indicators of neuroaxonal and astroglial injury in AGS, capturing neurological disease burden and treatment-associated changes more accurately than the IFN score. These findings support their use as objective biomarkers for monitoring immune-mediated brain injury and therapeutic response, and warrant validation in larger longitudinal studies.

## 1 Introduction

Aicardi–Goutières syndrome (AGS) is a rare, early-onset neuroinflammatory disorder characterized by chronic activation of antiviral type I interferon (IFN) signaling (1). It typically presents within the first year of life as a subacute encephalopathy, marked by irritability, dystonia, and developmental regression, often progressing to severe neurological impairment. Systemic inflammatory features, such as recurrent fever, autoimmune hepatitis, chilblain skin lesions, and cytopenias, may also be present. However, the clinical spectrum is broad, and milder phenotypes with later onset have been reported. The pathogenesis of AGS is driven by mutations in one of nine genes (AGS1–AGS9) involved in nucleic acid metabolism or sensing. As a consequence, the innate immune system is aberrantly activated (1).

Janus kinase (JAK) inhibitors, such as baricitinib and ruxolitinib, which block type I IFN signaling at the IFN-α receptor, have emerged as promising therapeutic options. Their use has been associated with reduced IFN activity and clinical improvement, supporting their potential to modulate IFN-mediated inflammation (2–4). However, the clinical benefit of JAK inhibition in AGS remains debated. While studies have demonstrated improvements in cutaneous and systemic symptoms, effects on neurological outcomes are inconsistent. This has been attributed to limited central nervous system (CNS) drug penetration and delayed treatment initiation (2–4). Currently, the IFN signature, determined by the expression of IFN-stimulated genes in blood, is used as a biomarker for AGS. While the IFN score represents a well-established and clinically valuable peripheral biomarker in type I interferonopathies, it can also be influenced by physiological responses to infection and does not directly reflect neurological damage, which may limit its utility for assessing neurological disease severity or treatment response. Although the IFN score shows strong correlations with systemic inflammatory conditions such as lupus or systemic sclerosis (5, 6), its ability to predict neurological damage in AGS remains unknown. Moreover, assessing neurological improvement in AGS is particularly challenging, as current evaluations rely primarily on observable clinical changes rather than direct, quantitative measures of neurological function.

Neurofilament light chain (NfL) has emerged as a highly sensitive blood-based biomarker of neuronal injury across diverse neurological disorders, including multiple sclerosis and amyotrophic lateral sclerosis (7). Its clinical utility is well established for assessing disease activity, monitoring progression, and evaluating treatment response, and it is increasingly applied as an outcome measure in clinical trials. Glial fibrillary acidic protein (GFAP) is an astrocyte-derived intermediate filament released upon astroglial activation and injury. Circulating GFAP has emerged as a sensitive biomarker of astroglial pathology across neurological disorders (8). In contrast to pNfL, which reflects neuroaxonal damage, pGFAP provides complementary information on astrocytic and neuroinflammatory responses, together capturing distinct but interconnected aspects of CNS injury. We therefore investigated whether plasma NfL (pNfL) levels can capture disease activity and the therapeutic response to JAK inhibition in AGS.

## 2 Methods

### 2.1 Study design and participants

This retrospective observational study included plasma samples from patients with genetically confirmed Aicardi–Goutières syndrome (AGS; n = 55) and age- and sex-matched healthy individuals (n = 55), ranging in age from 0 to 42 years. Cross-sectional analyses were performed using all available samples. Individuals were classified as healthy if they had no known acute or chronic illness at the time of blood collection. For a subset of patients, only plasma samples obtained during treatment with Janus kinase (JAK) inhibitors were available; these patients were included in cross-sectional comparisons according to treatment status. In addition, longitudinal analyses were performed in patients with paired plasma samples collected before and during JAK inhibitor therapy. Disease severity was classified based on age at onset of neurological symptoms, with onset before 9 months of age defined as severe and onset after 9 months with residual gross motor development defined as mild. Detailed clinical characteristics of all patients are provided in **Supplementary Table 1**.

### 2.2 Single molecule array

Plasma concentrations of neurofilament light chain (NfL) and glial fibrillary acidic protein (GFAP) were measured by Single Molecule Array (Simoa^®^) technology using the Neurology 2-Plex B (GFAP, NfL) Advantage Assay Kit on the Quanterix HD-X platform (Quanterix Corp., Billerica, MA, USA). Analyses were conducted according to the manufacturer’s instructions. Plasma samples were diluted in buffer (1:4) prior to measurement and processed as previously described (9).

### 2.3 Interferon signature

Gene expression of selected IFN-stimulated genes was analyzed to assess the presence of an IFN signature in blood. Peripheral blood mononuclear cells (PBMCs) were isolated from heparinized blood by Ficoll gradient centrifugation. Total RNA was extracted from PBMCs using the ReliaPrep RNA Cell Miniprep System (Promega), followed by DNase I digestion. RNA was reverse-transcribed using the GoScript Reverse Transcription System (Promega). Gene expression was determined by quantitative real-time reverse transcription (RT)-PCR using the TaqMan Universal PCR Master Mix (Applied Biosystems) on an ABI7300 and normalized to *GAPDH* (forward: GAAGGTGAAGGTCGGAGTC; reverse: GAAGATGGTGATGGGATTTC) and hypoxanthine phosphoribosyltransferase 1 (Hs02800695_m1, Thermo Fisher Scientific) expression. For calibration, a calibrator cDNA was included in each assay. Target genes were analyzed using predesigned TaqMan probes (Thermo Fisher Scientific) for *IFI27* (Hs01086373_g1), *IFI44* (Hs00951349_m1), *IFI44L* (Hs00915292_m1), *IFIT1* (Hs01675197_m1), *ISG15* (Hs01921425_s1), *RSAD2* (Hs01057264_m1), and *SIGLEC1* (Hs00988063_m1). The IFN score was calculated as previously described (10).

### 2.4 Statistical analysis

Statistical analyses were performed using Microsoft Excel and GraphPad Prism version 10.4.1. Continuous and ordinal variables were summarized as medians with interquartile ranges (IQR), while categorical variables were presented as absolute counts and percentages. Scatter plots depicting NfL and GFAP levels, as well as IFN scores, were generated in relation to age and sex. Normality of the datasets was assessed using the Shapiro-Wilk test and quantile-quantile (Q-Q) plots. Associations between pNfL, pGFAP, and IFN scores were evaluated using Spearman’s rank correlation. Only patients with available measurements for all three biomarkers (NfL, GFAP, and IFN score) were included in the analyses. Differences in pNfL and pGFAP levels across age groups relative to controls were analyzed using two-way ANOVA. For datasets that did not meet normality assumptions, comparisons between two independent groups were conducted using the Mann-Whitney-U test or Kruskal-Wallis test. Paired data, such as pre- and post-treatment values within the same individual, were analyzed using the Wilcoxon signed-rank test. Two-sided *P* values < 0.05 were considered statistically significant. Confidence intervals (95%) for median differences and Spearman correlation coefficients were estimated using bootstrap resampling (10,000 iterations). For each analysis, samples were repeatedly resampled with replacement to generate empirical distributions of the statistics, from which percentile-based confidence intervals were derived. This non-parametric approach was chosen to avoid distributional assumptions and to ensure robust estimation given the skewed nature of the data.

## 3 Results

### 3.1 Elevated plasma NfL and GFAP levels in patients with Aicardi–Goutières syndrome

We conducted a cross-sectional analysis of plasma samples from 55 patients with genetically confirmed AGS and 55 age- and sex-matched controls; detailed clinical and genotype information is provided in **Supplementary Table 1**. Plasma NfL levels were quantified using single molecule array technology, and plasma levels of glial fibrillary acidic protein (GFAP), a marker of astroglial activation and injury, were measured in parallel.

**Figure 1:**
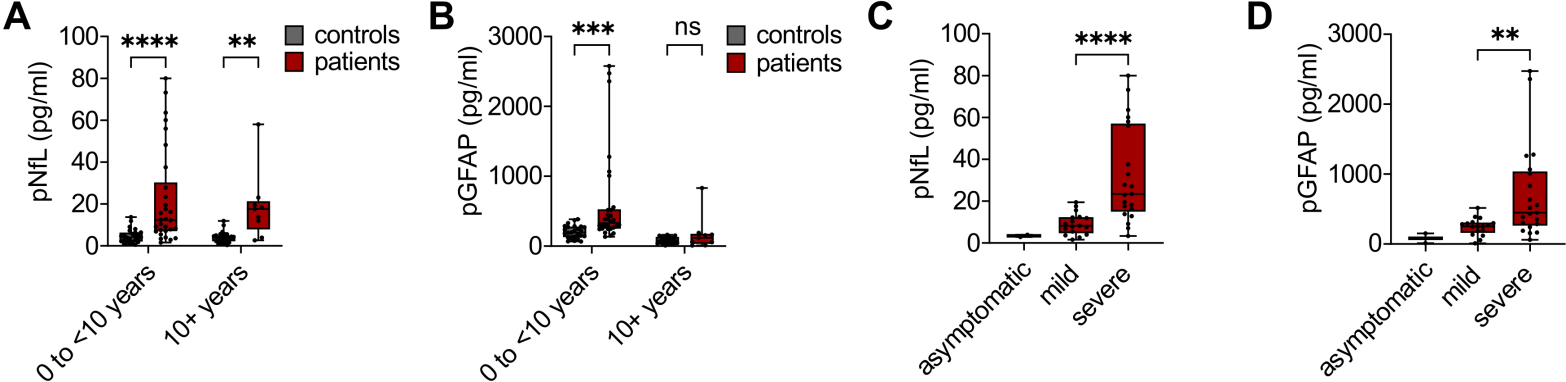
Cross-sectional analysis of plasma NfL and GFAP levels in AGS patients. pNfL **(A)** and pGFAP **(B)** levels in treatment-naïve AGS patients aged 0 to <10 years (group 1, n = 30) and ≥10 years (group 2, n = 9) compared with age-matched healthy controls (group 1, n = 25; group 2, n = 30). pNfL **(C)** and pGFAP **(D)** levels in treatment-naïve AGS patients stratified by clinical severity (asymptomatic, n = 2; mild, n = 17; severe, n = 21). For patients with multiple measurements, mean values were used. Statistical analyses for age-group comparisons were performed using two-way ANOVA with uncorrected Fisher’s least significant difference test. ****P < 0.0001 for pNfL patients vs. controls in group 1. **P < 0.007 for pNfL patients vs. controls in group 2. ***P < 0.0003 for pGFAP patients vs. controls in group 1. *P* = 0.5446 for pGFAP patients vs. controls in group 2. Severity comparisons were performed using the Mann–Whitney U test. ****P < 0.0001 for pNfL mild vs. severe. **P < 0.0036 for pGFAP mild vs. severe. Boxplots depict the 25^th^ and 75^th^ percentiles, center lines indicate medians, and whiskers represent minimum to maximum values.

Among treatment-naïve AGS patients stratified by age, pNfL levels were significantly higher than in controls in both the 0 to <10 years (median controls 4.16 pg/mL [interquartile range 2.08-6.34] vs. patients 12.32 pg/mL [7.05-30.24], median difference 8.16 pg/mL, 95% CI 3.54–14.88, p<0.0001), and the ≥10 years age groups (median controls 3.82 pg/mL [2.23-5.00] vs. patients 17.64 pg/mL [7.90-21.28], median difference 13.82 pg/mL, 95% CI 0.47–18.82, p<0.007) (**Figure 1A**). pGFAP levels were significantly elevated in patients younger than 10 years (median controls 194.6 pg/mL [129.74-277.86] vs. patients 332.2 pg/mL [250.43-526.40], median difference 137.60 pg/mL, 95% CI 48.82–259.72, p<0.0003), but not in the older age group (median difference 44.14 pg/mL, 95% CI −62.08 to 108.98) (**Figure 1B**), which may be attributed to the smaller sample size in the older age group. Both pNfL and pGFAP levels tended to be higher in younger patients, who more often represent with earlier disease onset and more severe clinical manifestations, consistent with ongoing neuroaxonal injury in this age group in AGS. Importantly, biomarker concentrations observed in the control cohort were within the reference ranges reported in previous pediatric and adult studies (8, 11, 12), supporting the validity of the measurements.

### 3.2 pNfL and pGFAP reflect clinical disease severity and dissociate from the interferon score

To assess the clinical relevance of pNfL and pGFAP, we next examined their relationship with disease severity and the IFN score. Disease severity was classified based on age at onset of neurological symptoms, with onset before 9 months defined as severe and onset after 9 months with residual gross motor development defined as mild.

Both pNfL and pGFAP levels correlated with clinical disease severity, with significantly higher concentrations observed in patients with severe disease compared to those with milder symptoms (**Figures 1C, D**). These findings indicate that markers of neuroaxonal and astroglial injury reflect the extent of neurological involvement in AGS.

**Figure 2:**
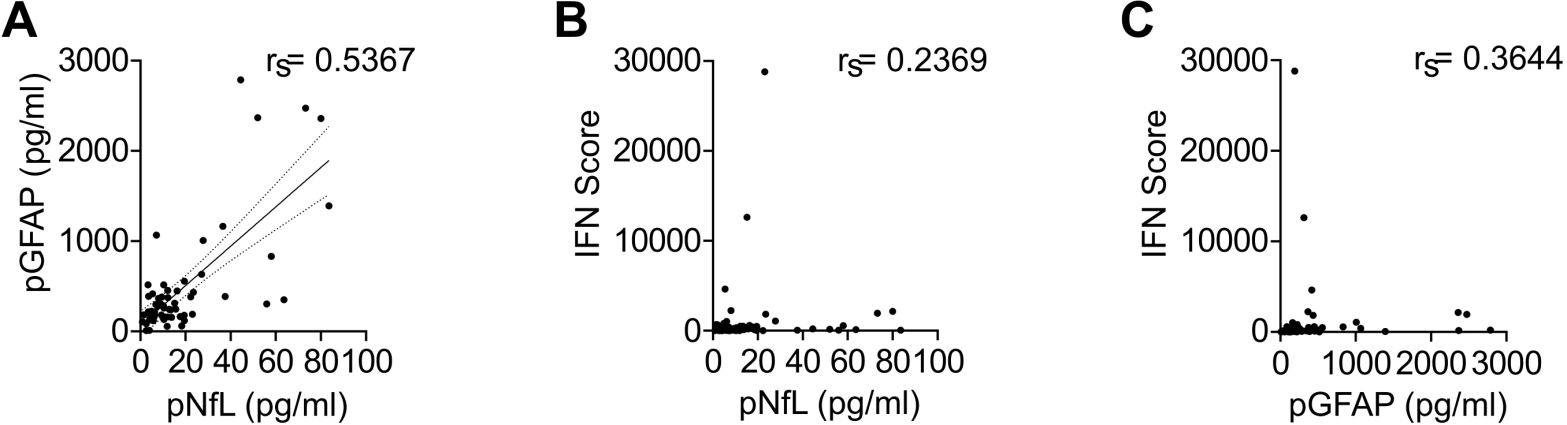
Correlation of neuroaxonal and astroglial injury markers with the interferon score in in AGS patients. Correlation analyses in treatment-naïve AGS patients showing the relationship between pNfL and pGFAP **(A)**, pNfL and the interferon (IFN) score **(B)**, and pGFAP and the IFN score **(C)**. Correlations were assessed using Spearman’s rank correlation coefficient.

Correlation analysis revealed a strong positive correlation between pNfL and pGFAP levels (Spearman´s r_s_ = 0.5367, p < 0.0001), consistent with concurrent neuroaxonal and astrocytic injury (**Figure 2A**). In contrast, pNfL showed only a weak correlation with the IFN score (r_s_ = 0.2369, p = 0.07), and pGFAP showed a moderate correlation (r_s_ = 0.3644, p = 0.05) (**Figures 2B, C**). This dissociation suggests that the peripheral IFN signature primarily captures systemic immune activation, which can also occur during intercurrent infections, and does not reliably reflect the degree of neurological damage.

### 3.3 JAK inhibitor therapy reduces pNfL and pGFAP but not the IFN score

We next examined the effect of immunosuppressive therapy with Janus kinase (JAK) inhibitors on plasma biomarker levels. In cross-sectional analyses stratified by treatment status, untreated AGS patients displayed significantly higher pNfL and pGFAP levels than healthy controls, whereas treated patients exhibited intermediate values (**Supplementary Figure 1**). Although biomarker concentrations in treated patients were lower than in untreated patients, these differences did not reach statistical significance, likely reflecting inter-individual variability and limited sample size.

**Figure 3:**
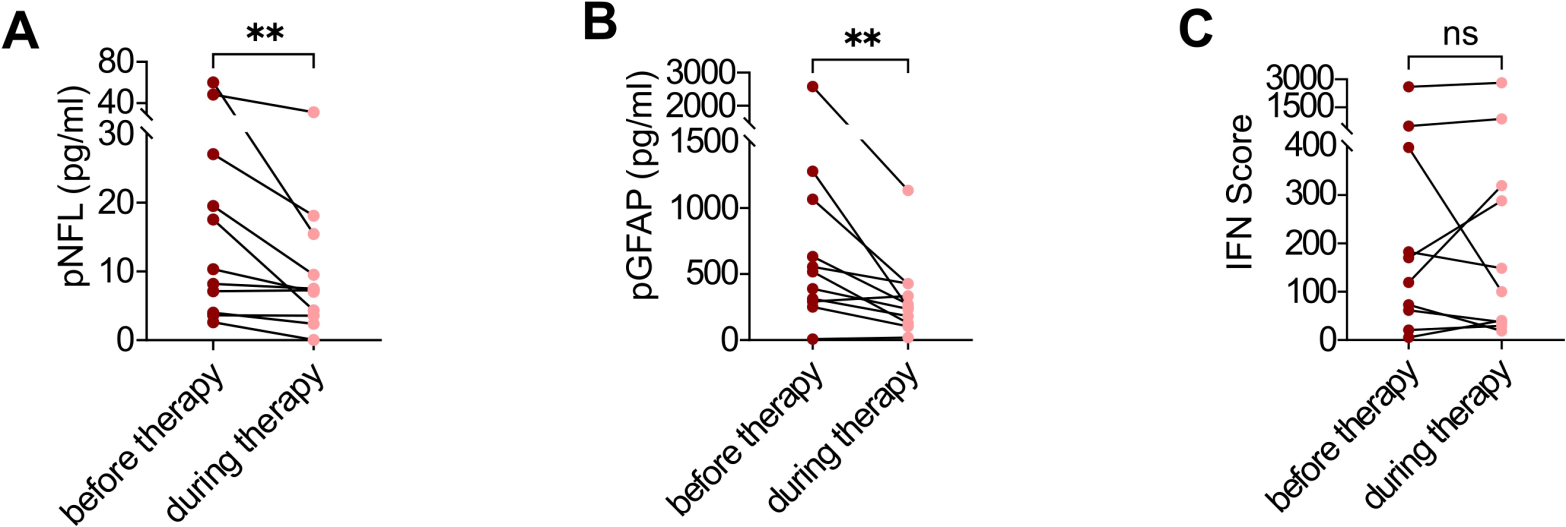
Longitudinal effects of JAK inhibitor therapy on plasma NfL, GFAP, and the interferon score. Longitudinal within-patient analysis of plasma pNfL **(A)**, pGFAP **(B)**, and IFN score **(C)** in AGS patients before and during JAK inhibitor treatment (n = 11). For each patient, measurements before and during therapy were averaged. Statistical comparisons were performed using the Wilcoxon signed-rank test. **P = 0.0029 for pNfL, **P = 0.0049 for pGFAP, and *P* = 0.4316 for IFN score for comparison before vs. during therapy.

To directly assess treatment effects independent of between-patient heterogeneity, we performed longitudinal analyses in a subset of 11 patients with paired samples obtained before and after initiation of JAK inhibitor therapy. Patients received JAK inhibitor therapy with heterogeneous dosing regimens and treatment durations, reflecting real-world clinical practice (**Supplementary Table 2**). In this within-patient comparison, both pNfL and pGFAP levels declined significantly following treatment (median pNfL before therapy 10.32 pg/mL [3.992–27.08] vs. during therapy 7.28 pg/mL [3.57–15.46], *p* < 0.0029; median pGFAP before therapy 516.8 pg/mL [293.1–1066.0] vs. during therapy 248.3 pg/mL [128.0–424.0], *p* < 0.0049), demonstrating a clear treatment-associated reduction in markers of neuroaxonal and astroglial injury (**Figures 3A, B**). This corresponded to a median decrease in pNfL of 3.28 pg/mL (95% CI 0.69 to 13.20) and in pGFAP of 152.72 pg/mL (95% CI 128.54 to 642.00) after initiation of JAK inhibitor therapy, whereas the IFN score did not show a consistent change (**Figure 3C**).

## 4 Discussion

Our primary objective was to determine whether plasma NfL and GFAP provide a more robust indicator of disease activity than the IFN score in AGS, with a particular focus on neurological involvement. Neurological assessment in young children is particularly challenging, as developmental variability and the dynamic nature of neurodevelopment particular in early childhood complicate the use of standardized, quantitative outcome measures. Moreover, in this age group, it is often difficult to discern whether observed clinical changes reflect natural developmental progress or the effect of a therapeutic intervention. In this context, objective biomarkers such as pNfL and pGFAP are of particular value, as they provide quantitative parameters of neuronal and glial injury that are independent of clinical scoring systems and observer bias (7).

Our findings demonstrate that both pNfL and pGFAP are markedly elevated in untreated AGS patients, correlate with clinical disease severity, and outperform the IFN score in reflecting neurological involvement. The strong correlation between pNfL and pGFAP suggests that neuroaxonal damage and astroglial activation occur in parallel in AGS, consistent with an ongoing neuroinflammatory process. In contrast, the weak association between these biomarkers and the IFN score indicates that systemic interferon activity does not reliably capture the extent of CNS injury. In contrast to the IFN score, both pNfL and pGFAP more directly reflect neurological tissue injury and provide complementary information on disease burden. Together, pNfL and pGFAP may provide a more comprehensive view of neuroaxonal and astroglial injury in interferonopathies. This limitation is further underscored by the fact that the IFN score is not disease-specific and can be transiently elevated during common viral infections, reflecting physiological antiviral responses rather than AGS-related pathology. As a result, IFN signatures may be confounded by intercurrent infections and systemic immune activation, diminishing their reliability as biomarkers for neurological disease burden or treatment response in interferonopathies.

Importantly, longitudinal within-patient analyses revealed significant reductions in pNfL and pGFAP following initiation of JAK inhibitor therapy, whereas cross-sectional comparisons between treated and untreated patients did not reach statistical significance. This discrepancy likely reflects substantial inter-individual variability, disease heterogeneity, and limited sample size, rather than an absence of treatment effect. By minimizing between-patient confounders, longitudinal analyses provide a more sensitive framework for detecting therapy-associated changes and underscore the value of pNfL and pGFAP as dynamic biomarkers for monitoring neurological responses to immunosuppressive treatment in AGS.

The observed association between declining pNfL and pGFAP levels and JAK inhibitor therapy aligns with emerging evidence that extracellular NfL is not merely a passive marker of axonal damage but may actively contribute to myeloid cell activation and neuroinflammation (13). This dual role positions NfL as both a readout and a potential amplifier of immune-mediated neuronal injury. Accordingly, reductions in circulating pNfL and pGFAP under immunosuppressive therapy likely reflect attenuation of ongoing neuroinflammatory processes rather than solely reduced downstream tissue damage, thereby providing a biological correlate of clinical improvement.

This study has several limitations. The sample size is limited, particularly for subgroup and longitudinal analyses. In addition, the retrospective design and heterogeneity of clinical follow-up may introduce variability in sampling and clinical assessment. Finally, standardized neurological outcome measures were not systematically available, which limits direct correlation of biomarker dynamics with clinical progression. In addition, disease severity was classified based on age at onset, as detailed neurological parameters were not consistently available across the cohort, potentially limiting the granularity of severity classification.

Taken together, these findings establish pNfL and pGFAP as sensitive, easily accessible biomarkers of neurological involvement and therapeutic response in AGS. Their incorporation into future clinical studies could facilitate patient stratification, enable objective assessment of neurological outcomes, and improve the evaluation of immunomodulatory therapies in type I interferonopathies. Larger longitudinal studies will be required to validate their performance for long-term disease monitoring, to define clinically meaningful thresholds, and to assess their utility in guiding individualized treatment decisions.

## Data Availability

The original contributions presented in the study are included in the article/**Supplementary Material**. Further inquiries can be directed to the corresponding author.
