# Supplementary material for: Beyond the interferon score: neurofilament light chain and glial fibrillary acidic protein capture immune-mediated neuroinjury and response to JAK inhibition in Aicardi–Goutières syndrome
Source: Front Immunol. 2026 Apr 15;17:1782352. doi: 10.3389/fimmu.2026.1782352 (PMC13124696; doi:10.3389/fimmu.2026.1782352)
Supplement: Supplementary file 1 [file SupplementaryFile1.docx]

Supplementary Material

## Supplementary Table 1. Clinical findings in AGS patients

| **AGS subtype** | **Gene** | **Mutation** | **Sex** | **Age at onset** | **Clinical findings** | **Current treatment** |
| --- | --- | --- | --- | --- | --- | --- |
| AGS1 | TREX1 | c.341G>T; p.Arg114Leu (hom) | M | 3 y | chilblains | none |
| AGS1 | TREX1 | c.341G>T; p.Arg114Leu (hom) | M | 2 m | tetraspasticity, hepatopathy, global developmental delay | ruxo |
| AGS1 | TREX1 | c.401_408dup, p.Ser137fs (hom) | F | 2 m | tetraspasticity, hepatopathy, leukodystrophy, microcephaly, global developmental delay | ruxo |
| AGS1 | TREX1 | c.143_144del; p.Pro48fs, c.294_296dup; p.Cys99Ter | M | 2 wk | dystonia, tetraspasticity, microcephaly, global developmental delay | ruxo |
| AGS1 | TREX1 | c.868_885del; p.Pro290_Ala295del, c.907A>C; p.Thr303Pro | F | 2 m | dystonia, tetraspasticity, microcephaly, global developmental delay | none |
| AGS1 | TREX1 | c32T>G; p.Met11Arg, c.341G>A; p.Arg114His | F | neonatal | dystonia, tetraspasticity, thrombocytopenia, microcephaly, leukodystrophy, blindness, global developmental delay | none |
| AGS2 | RNASEH2B | c.436+1G>A; ?, c.529G>A; p.Ala177Thr | M | 6 m | tetraspasticity, global developmental delay, microcephaly | ruxo |
| AGS2 | RNASEH2B | c.529G>A; p.Ala177Thr, c.634G>A; p.Ala212Thr | M | > 12 m | spastic paraparesis, able to walk and talk | ruxo |
| AGS2 | RNASEH2B | c.529G>A, p.Ala177Thr (hom) | F | 18 m | spastic paraparesis, able to walk and talk | none |
| AGS2 | RNASEH2B | c.529G>A, p.Ala177Thr (hom) | F | n/a | asymptomatic | none |
| AGS2 | RNASEH2B | c.136+1del; ?; c.529G>A, p.Ala177Thr | F | 6 m | dystonia, tetraspasticity, global developmental delay | ruxo |
| AGS2 | RNASEH2B | c.529G>A, p.Ala177Thr (hom) | M | 9 m | spastic paraparesis, chilblains, able to talk | bari |
| AGS2 | RNASEH2B | c.529G>A, p.Ala177Thr (hom) | M | 1 m | dystonia, tetraspasticity, sensory deafness, global developmental delay | none |
| AGS2 | RNASEH2B | c.529G>A, p.Ala177Thr (hom) | M | 3 m | dystonia, tetraspasticity, seizures, leukodystrophy, microcephaly, global developmental delay | ruxo |
| AGS2 | RNASEH2B | c.529G>A, p.Ala177Thr (hom) | F | 9 m | spastic paraparesis, gross motor developmental delay | none |
| AGS2 | RNASEH2B | c.529G>A, p.Ala177Thr (hom) | M | 17 m | spastic paraparesis | ruxo |
| AGS2 | RNASEH2B | c.469A>G; p.Lys157Glu, c.476G>T; p.Ser159Ile | M | 11 m | spastic paraparesis | none |
| AGS5 | SAMHD1 | c.433C>T; p.Arg145Ter (hom) | M | neonatal | tetraspasticity, hepatopathy, leukodystrophy, seizures, microcephaly, global developmental delay | none |
| AGS5 | SAMHD1 | c.427C>T; p.Arg143Cys (hom) | F | neonatal | floppy infant, leukodystrophy, tetraspasticity, global developmental delay | none |
| AGS5 | SAMHD1 | c.626-1G>C; p.?, c.853-2A>G; p.? | F | 10 m | chilblains, dermatomyositis | bari |
| AGS5 | SAMHD1 | c.869G>A; p.Arg290His, c.1642C>T, p.Gln548Ter | M | 2 m | dystonia, tetraspasticity, microcephaly, global developmental delay, lupus, arthritis, chilblains | ruxo |
| AGS5 | SAMHD1 | c.434G>A; p.Arg145GIn, c.508G>A; p.Gly170Arg | F | 3 m | dystonia, tetraspasticity, microcephaly, global developmental delay, recurrent fever | none |
| AGS5 | SAMHD1 | c490C>T; p.Arg164Ter, c.817del; p.Val273Ter | M | 3 m | tetraspasticity, cerebrovascular disease, lupus panniculitis, lymphoma | none |
| AGS5 | SAMHD1 | c.490C>T; p.Arg164Ter, c.817del; p.Val273Ter | F | 3 m | tetraspasticity, cerebrovascular disease, lupus panniculitis, lymphoma | none |
| AGS6 | ADAR | c.3364A>C; p.Lys1122Gln (hom) | F | 2 m | tetraspasticity, global developmental delay | ruxo |
| AGS6 | ADAR | c.577C>G; p.Pro193Ala, c.3125delG; p.Arg1042fs | M | 3 m | tetraspasticity, dystonia, brain atrophy, global developmental delay | bari |
| AGS6 | ADAR | c.3019G>A; p.Gly1007Arg (het) | F | 10 m | gross motor developmental delay, able to walk | none |
| AGS6 | ADAR | c.3019G>A; p.Gly1007Arg (het) | F | 12 m | seizures, dystonia, sterile meningitis | none |
| AGS6 | ADAR | c.3019G>A; p.Gly1007Arg (het) | F | n/a | asymptomatic | none |
| AGS6 | ADAR | c.665_666insG; p.Asn222fs (het, de novo) | F | 2 m | dystonia, tetraspasticity, seizures, global developmental delay, cardiomyopathy, microcephaly | none |
| AGS6 | ADAR | c.577C>G, p.Pro193Ala, c.3149G>A; p.Gly1050Glu | M | 12 m | dystonia, spastic paraparesis, able to walk | none |
| AGS6 | ADAR | c.518A>G; p.Asn173Ser, c.1379del; p.Pro460fs | M | 9 m | spastic paraparesis, gross motor developmental delay | ruxo |
| AGS6 | ADAR | c.1A>G; p.Met1?, c.577C>G; p.Pro193Ala) | F | 13 m | dystonia, spastic paraparesis, able to talk and write, chilblains | none |
| AGS6 | ADAR | c.1A>G; p.Met1?, c.577C>G; p.Pro193Ala) | M | 3 y | transient encephalopathy, chilblains | none |
| AGS6 | ADAR | c.3019G>A; p.Gly1007Arg (het) | M | 3 y | spastic paraparesis, able to walk and talk | ruxo |
| AGS6 | ADAR | c.3019G>A; p.Gly1007Arg (het) | F | 3 y | mild spastic paraparesis, able to walk and talk | none |
| AGS6 | ADAR | c.3019G>A; p.Gly1007Arg (het) | M | n/a | asymptomatic | none |
| AGS6 | ADAR | c.577C>G; p.Pro193Ala, c.1832del; p.Ser611fs | M | 3 m | dystonia, tetraspasticity, microcephaly, leukodystrophy, global developmental delay | ruxo |
| AGS6 | ADAR | c.577C>G; p.Pro193Ala, c.2433_2434del; p.Ala813fs | F | 9 m | spastic paraparesis, able to talk | ruxo |
| AGS6 | ADAR | c.3019G>A; p.Gly1007Arg (het, de novo) | M | 9 m | spastic paraparesis | ruxo |
| AGS6 | ADAR | c.982C>T; p.Arg328Ter (het) | M | 10 m | spastic paraparesis | ruxo |
| AGS7 | IFIH1 | c.2336G>A; p.Arg779His (het) | F | > 12 m | dystonia, spasticity, able to sit and walk with support | ruxo |
| AGS7 | IFIH1 | c.2336G>A, p.Arg779His (het) | F | 1 m | dystonia, tetraspasticity, microcephaly, global developmental delay | ruxo |
| AGS7 | IFIH1 | c.2561T>A; p.Met854Lys (het) | F | 4 m | dystonia, tetraspasticity, microcephaly, global developmental delay | none |
| AGS7 | IFIH1 | c.2156C>T; p.Ala719Val (het, de novo) | F | 2 m | dystonia, tetraspasticity, microcephaly, global developmental delay, autoimmune hepatitis | ruxo |
| AGS7 | IFIH1 | c.1246A>C; p.Ile416Leu (het, de novo) | M | 9 m | spastic paraparesis, able to talk | ruxo |
| AGS7 | IFIH1 | c.2336G>A; p.Arg779His (het, de novo) | M | 3 m | dystonia, tetraspasticity, microcephaly, global developmental delay | none |
| AGS7 | IFIH1 | c.2465G>A; p.Arg822Gln (het, de novo) | M | 18 m | unsteady gait, recurrent fever, arthritis, enthesitis, dystrophy, normal mental development | bari |
| AGS7 | IFIH1 | c.2518G>A; p.Val840lle (het, de novo) | M | 3 m | dystonia, tetraspasticity, microcephaly, leukodystrophy, global developmental delay | ruxo |
| AGS7 | IFIH1 | c.2336G>A; p.Arg779His (het) | M | 15 m | spastic paraparesis, able to walk and talk | ruxo |
| AGS7 | IFIH1 | c.2336G>A; p.Arg779His (het) | M | 16 m | spastic paraparesis, able to walk and talk | ruxo |
| AGS7 | IFIH1 | c.1873C>T; p.His625Tyr (het, de novo) | M | 3 m | dystonia, tetraspasticity, microcephaly, leukodystrophy, global developmental delay | none |
| AGS7 | IFIH1 | c.2159G>A; p.Arg720Gln (het, de novo) | M | neonatal | hepatitis, seizures, thrombocytopenia, leukodystrophy | ruxo |
| AGS9 | RNU7-1 | n.30A>G (hom) | F | 11 m | dystonia, spastic paraparesis | ruxo |
| AGS9 | RNU7-1 | n.30A>G, ก.40_47delCTGGCTTT | M | 10 m | spastic paraparesis, able to walk and talk | none |

Hom, homozygous; het, heterozygous; F, female; M, male; m, month; wk, week; y, year; n/a, not applicable; ruxo, ruxolitinib; bari, baricitinib.

## Supplementary Table 2. JAK inhibitor treatment characteristics in longitudinally analyzed AGS patients

| **Patient** | **AGS  subtype** | **JAK  inhibitor** | **Dose** | **Treatment duration (months)** |
| --- | --- | --- | --- | --- |
| 1 | AGS7 | Ruxolitinib | 0.5 mg/kg | 48 |
| 2 | AGS6 | Ruxolitinib | 0.5 mg/kg | 6 |
| 3 | AGS2 | Ruxolitinib | 10 mg/day | 30 |
| 4 | AGS2 | Ruxolitinib | 0.5 mg/kg | 60 |
| 5 | AGS2 | Baricitinib | 4 mg/day | 10 |
| 6 | AGS1 | Ruxolitinib | 0.5 mg/kg | 58 |
| 7 | AGS7 | Ruxolitinib | 0.5 mg/kg | 48 |
| 8 | AGS6 | Ruxolitinib | 0.5 mg/kg | 46 |
| 9 | AGS6 | Ruxolitinib | 0.5 mg/kg | 18 |
| 10 | AGS6 | Ruxolitinib | 0.5 mg/kg | 40 |
| 11 | AGS7 | Ruxolitinib | 2 mg/day | 36 |

Doses are reported either as weight-based (mg/kg) or fixed daily dosing, according to clinical practice.

## Supplementary Figure 1


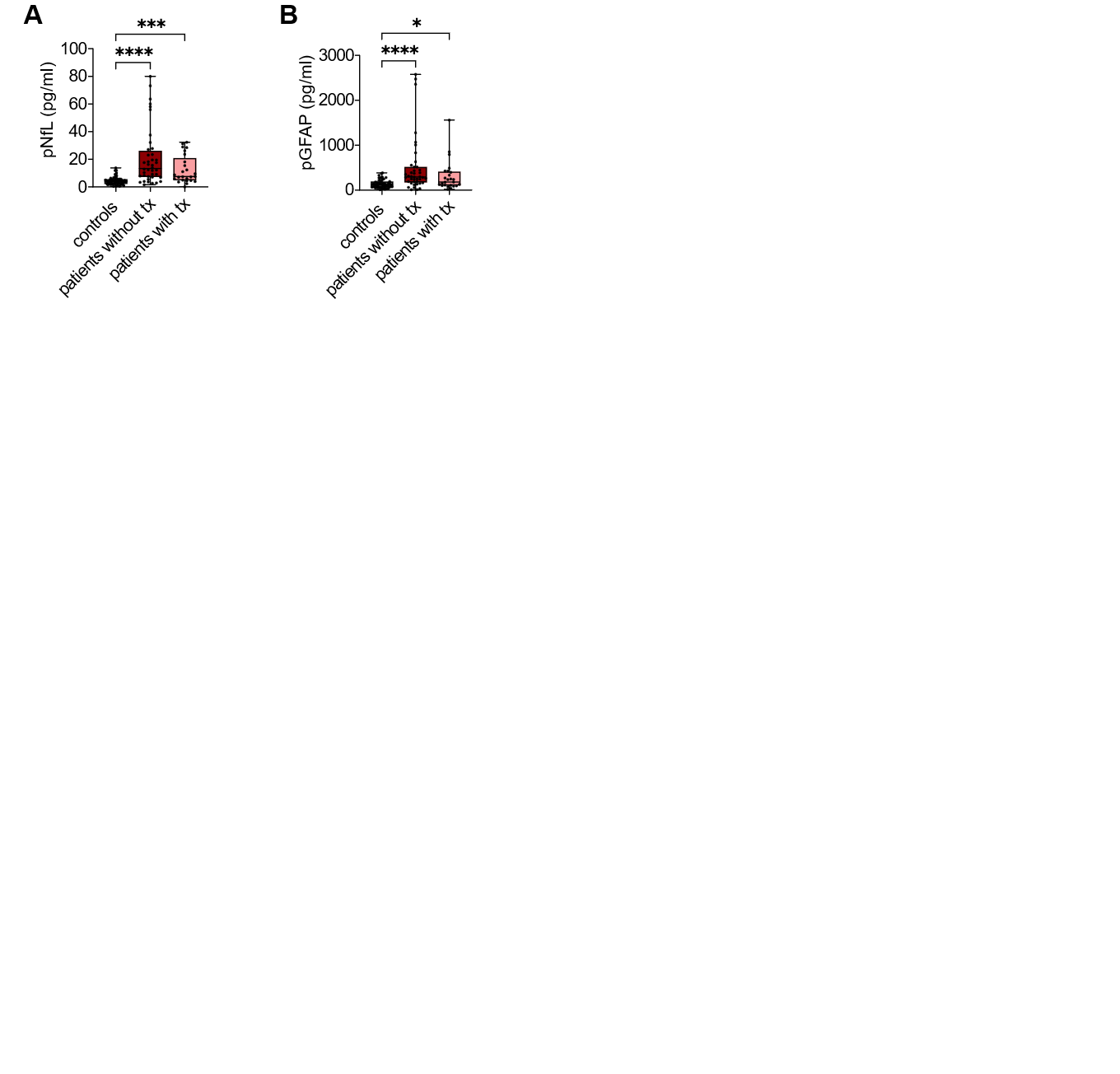


**Supplementary Figure 1. Cross-sectional comparison of plasma NfL and GFAP levels by treatment status.** (**A**) pNFL levels in healthy controls (n = 55), untreated AGS patients (n = 40), and patients receiving JAK inhibitor therapy (n = 25). (**B**) pGFAP levels in the same groups (controls, n = 55; untreated, n = 41; treated patients, n = 25). If patients had measurements both with and without therapy, they were assigned to each group accordingly. For patients with multiple measurements, the mean value was used. Statistical analyses were performed using the Kruskal–Wallis test. *****P* < 0.0001 for controls vs. untreated patients; *** *P* = 0.0001 for controls vs. treated patients; *P* = 0.4442 for untreated vs. treated patients (A). *****P* < 0.0001 for controls vs. untreated patients; **P* = 0.0256 for controls vs. treated patients; *P* = 0.1824 for untreated vs. treated patients (B). Boxplots depict the 25^th^ and 75^th^ percentiles, center lines indicate medians, and whiskers represent minimum to maximum values.
